# Nipah Virus-associated Encephalitis Outbreak, Siliguri, India

**DOI:** 10.3201/eid1202.051247

**Published:** 2006-02

**Authors:** Mandeep S. Chadha, James A. Comer, Luis Lowe, Paul A. Rota, Pierre E. Rollin, William J. Bellini, Thomas G. Ksiazek, Akhilesh C. Mishra

**Affiliations:** * National Institute of Virology, Pune, India;; † Centers for Disease Control and Prevention, Atlanta, Georgia, USA

**Keywords:** Research, India, Nipah virus, encephalitis

## Abstract

During January and February 2001, an outbreak of febrile illness associated with altered sensorium was observed in Siliguri, West Bengal, India. Laboratory investigations at the time of the outbreak did not identify an infectious agent. Because Siliguri is in close proximity to Bangladesh, where outbreaks of Nipah virus (NiV) infection were recently described, clinical material obtained during the Siliguri outbreak was retrospectively analyzed for evidence of NiV infection. NiV-specific immunoglobulin M (IgM) and IgG antibodies were detected in 9 of 18 patients. Reverse transcription–polymerase chain reaction (RT-PCR) assays detected RNA from NiV in urine samples from 5 patients. Sequence analysis confirmed that the PCR products were derived from NiV RNA and suggested that the NiV from Siliguri was more closely related to NiV isolates from Bangladesh than to NiV isolates from Malaysia. NiV infection has not been previously detected in India.

## Abstract 2

Nipah virus, not previously detected in India, caused an outbreak of febrile encephalitis in West Bengal.

During January and February of 2001, an outbreak of febrile illness with altered sensorium was observed in Siliguri, West Bengal, India. Siliguri is an important commercial center with a population of ≈500,000. It is near borders with China, Bangladesh, Nepal, and Sikkim. The outbreak occurred among hospitalized patients, family contacts of the patients, and medical staff of 4 hospitals. Japanese encephalitis, which is endemic in this area, was initially suspected, but the age group affected and the epidemiologic features suggested a different disease. Laboratory investigations conducted at the time of the outbreak failed to identify an infectious agent (*1*).

Nipah virus (NiV), a recently emergent, zoonotic paramyxovirus (*2*), was implicated as the cause of a highly fatal (case-fatality ratio 38%–75%), febrile human encephalitis in Malaysia and Singapore in 1999 (*1*) and in Bangladesh during the winters of 2001, 2003, and 2004 (*3**–**6*). The natural reservoir of NiV is presumed to be fruit bats of the genus *Pteropus*. Evidence of NiV infection was detected in these bats in Malaysia, Bangladesh, and Cambodia (*7**–**10*). In the Malaysian outbreak, NiV was introduced into the pig population, and most of the human cases resulted from exposure to ill pigs (*2*). However, an intermediate animal host was not identified during the Bangladesh outbreaks, which suggests that the virus was transmitted either directly or indirectly from infected bats to humans. Human-to-human transmission of NiV was also documented during the outbreak in Faridpur, Bangladesh (*4**,**5*). Because the clinical manifestations of the cases in Siliguri were similar to those of NiV cases in Bangladesh (*3**–**6*), and because Siliguri is near affected areas in Bangladesh, a retrospective analysis of clinical samples was undertaken to determine if NiV was associated with the Siliguri outbreak.

## Methods

### Case Definition and Clinical Sample Collection

A team of physicians and epidemiologists from the National Institute of Virology, Pune, India, along with local public health authorities, visited Siliguri. Investigations were conducted with the assistance of health authorities from West Bengal State and staff from the North Bengal Medical College Hospital. Medical records of patients who were hospitalized during the study period were examined, and their family members or caretakers were interviewed. Areas of the town in which cases occurred and the houses of patients who died were visited. Contacts and family members of patients who died were also interviewed.

A broad working case definition was used for case detection. The case definition evolved over the course of the outbreak on the basis of information from case-patients admitted to different hospitals, review of the line list of patients, and interviews with contacts in the community. A suspected patient was one >15 years of age with acute onset of high-grade fever and headache. A probable patient was one >15 years of age who had high-grade fever and altered sensorium and encephalitis of unknown origin. Blood samples were available for 18 hospitalized patients and for 13 family contacts of patients who died 2–3 weeks earlier. Six urine samples (5 samples had corresponding serum samples) were also collected.

### Serologic Testing

State health authorities conducted laboratory tests to rule out malaria and bacterial infections. Serologic tests to detect infection by Japanese encephalitis virus, West Nile virus, measles virus, dengue virus, *Leptospira* spp., and hantavirus were carried out at the National Institute of Virology.

Serum samples were gamma-irradiated at the Centers for Disease Control and Prevention (CDC) before being tested for immunoglobulin G (IgG) and IgM antibodies to NiV and measles by enzyme-linked immunosorbent assay (ELISA), as previously described (*2**,**11**,**12*). Briefly, inactivated antigens for these ELISAs were prepared from gamma (^60^Co)-irradiated NiV-infected or mock-infected Vero E6 cells. Serum samples were tested in 4-fold dilutions from 1:100 to 1:6,400. Samples were considered positive for the IgM assay if the sum of the adjusted optical densities (OD) from all of the dilutions (OD from infected antigen well minus OD from the mock-infected antigen) was >0.75 through the entire dilution series, and the titer was >1:400. Similarly, samples were considered positive for IgG if the sum for the adjusted OD from all the dilutions was >0.90 through the entire dilution series, and the titer was >1:400.

### Detection of NiV by RT-PCR and Virus Isolation

RNA was extracted from urine samples by using a Qiagen (Valencia, CA, USA) RNA extraction kit. Reverse transcription–polymerase chain reaction (RT-PCR) was performed with the SuperScript One-Step RT-PCR kit with Platinum Taq (Invitrogen, Carlsbad, CA, USA), as previously described (*13**,**14*). Reaction products were analyzed by agarose gel electrophoresis and ethidium bromide straining. PCR products were sequenced by using a cycle sequencing reaction with fluorescent dye terminators (Perkin-Elmer, Applied Biosystems Division, Foster City, CA, USA), and reaction products were analyzed with an ABI 3100 (Perkin-Elmer) automatic sequencer. Sequence data from multiple reactions were analyzed by using version 10.1 of the Genetics Computer Group Package (Accelrys, San Diego, CA, USA). Phylogenetic analyses were performed with PAUP version 4.01 (Sinauer Associates, Sunderland, MA, USA). Two sets of primers were used for RT-PCR reactions. Primer set NVNBF-4 (5´-GGAGTTATCAATCTAAGTTAG-3´) and NVNBR4 (5´-CATAGAGATGAGTGTAAAAGC-3´) amplified a 159-nucleotide (nt) region of the N gene of NiV. Primer set NVBMFC1 (5´-CAATGGAGCCAGACATCAAGAG-3´) and NVBMFR2 (5´-CGGAGAGTAGGAGTTCTAGAAG-3´) amplified a 320-nt region of the M gene. Virus isolation was attempted from the urine samples on Vero E6 cells as previously described (*2*).

## Results

**Figure 1:**
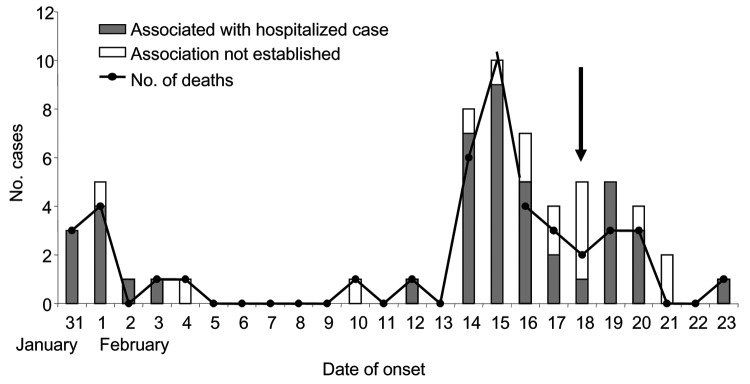
Epidemic curve of outbreak of febrile encephalitis in Siliguri, India, January though February 2001, by number of hospital-associated and nonhospital-associated cases and deaths. The vertical, black arrow indicates when barrier methods were introduced for case management.

**Figure 2:**
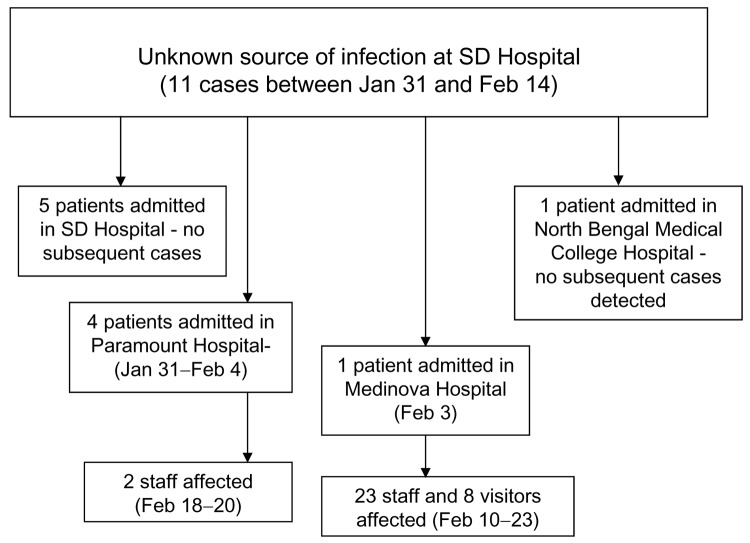
Sequence of events in the Siliguri (SD) outbreak.

The outbreak of fever with altered sensorium began in late January 2001 and peaked in mid-February. No cases were reported after February 23 (Figure 1). All of the patients were residents of Siliguri, and some clustering of cases was observed around the Medinova Hospital, since the staff of this hospital resided in the area. Based on the case definition, 66 cases of encephalitis were identified, and the case-fatality ratio was ≈74%. The outcome of 61 cases was known; the remaining 5 patients were discharged from the hospital against medical advice. All patients were >15 years of age; the male-to-female ratio was 1.4:1. Forty-five (75%) of the 60 patients had a history of hospital exposure, i.e., they were members of the hospital staff or had attended to or visited patients in the hospital. The outbreak began at a single hospital, and cases were subsequently detected at 3 other hospitals. No definitive information about the possible index case exists. Five families had >1 case, but all of the persons affected had a history of hospital visits and had onset of illness 2 or 3 days apart from each other. The sequence of events is shown in Figure 2.

The patients initially had fever (100%), headache and myalgia (57%), vomiting (19%), altered sensorium (confusion to coma, 97%), respiratory symptoms (tachypnea to acute respiratory distress, 51%), and involuntary movements or convulsions (43%). No neck rigidity or cranial nerve involvement was observed in the 16 patients who were examined. Pupils were bilaterally dilated and reactive to light. Deep tendon reflexes were diminished or absent. Abnormal plantar reflexes (extensor plantar response) were elicited in 11 patients. Patients were normotensive at admission but became hypertensive before death. Death occurred within 1 week of onset of disease in 10 patients (62.5%), within 2 weeks in 5 (32.8%) patients, and on day 30 after onset for 2 patients.

Before the outbreak, the staff did not routinely use personal protective equipment or barrier nursing methods. Use of surgical masks was minimal on wards, except in the intensive-care units. Certain universal precautions, such as hand washing and use of gloves, were partially adhered to when staff were carrying out invasive procedures. Patients were housed on wards with >4 patients in a single room and could be visited or be attended to by their family and others. Patients did not wear masks on wards or when being transported for procedures (e.g., x-ray examination). Disposal of waste, collection of soiled linen, laundry, and cleaning of floors and other surfaces in the wards were carried out by personnel who did not follow infection control practices.

Once the outbreak of encephalitis was established, stringent infection control practices were introduced (Figure 1), including isolating patients in the Medical College Hospital, where 2 wards were established, one for suspected and the other for probable cases. Barrier nursing techniques were initiated, and housekeeping procedures and waste management were improved.

Cerebrospinal fluid was obtained from all patients. Analysis showed that the fluid in all cases was under pressure and clear with <5 lymphocytes/mm^3^ (normal range 0–5 cells/mm^3^). These samples were not available for further analysis.

Laboratory testing during and immediately after the outbreak did not identify a likely etiologic agent. Patient serum samples were tested for IgM antibodies to Japanese encephalitis, West Nile, dengue, and measles viruses as well as for *Leptospira* spp. Serum samples were also tested for IgG antibody to hantavirus. All serologic tests were negative, and no likely viral or bacterial agents were detected. All serum samples tested positive for IgG to measles virus.

Because NiV was identified as the cause of encephalitis outbreaks in Bangladesh, the Siliguri samples were tested for evidence of NiV infection. In all, 17 serum samples were available from 18 patients from Siliguri. All were tested for IgG and IgM antibodies to NiV by ELISA. The 6 urine samples collected from these 18 patients were tested for NiV RNA by RT-PCR, and aliquots were inoculated onto Vero E6 cells in an attempt to isolate NiV.

NiV-specific IgM and IgG were detected in 9 of 17 serum samples; 1 sample was positive for IgG and negative for IgM (Table). RT-PCR assays detected RNA from the N gene of NiV in 4 urine samples from NiV antibody–positive patients and in 1 urine sample from a NiV antibody–negative patient. RNA from the M gene was detected in 3 of these 5 samples (Table). No viral isolates were obtained from the 6 urine samples.

**Table:** Serologic and PCR test results for clinical material from patients with encephalitis, Siliguri, India*

Patient no.	Days after onset of fever	Serology†		PCR (urine)	
		IgM	IgG	N gene	M gene
1	10	+	+	+	NA
2	5	+	+		NA
3	9	+	+	+	NA
4	10	+	+	NA	NA
5	9	–	–	NA	NA
6	10	–	–	+	+
7	3	–	–	NA	NA
8	7	–	–	NA	NA
9	Unknown	–	–	NA	NA
10	1	–	+	NA	NA
11	3	+	+	NA	NA
12	5	+	+	+	+
13	7	–	–	NA	NA
14	6	+	+	NA	NA
15	3	+	+	NA	NA
16	8	–	–	NA	NA
17	8	+	+	+	+
18	2	NA†	NA	–	–

*PCR, polymerase chain reaction; IgM, immunoglobulin M; IgG, immunoglobulin G; NA, no sample available. †Nipah virus–specific IgM or IgM by enzyme-linked immunosorbent assay.

**Figure 3:**
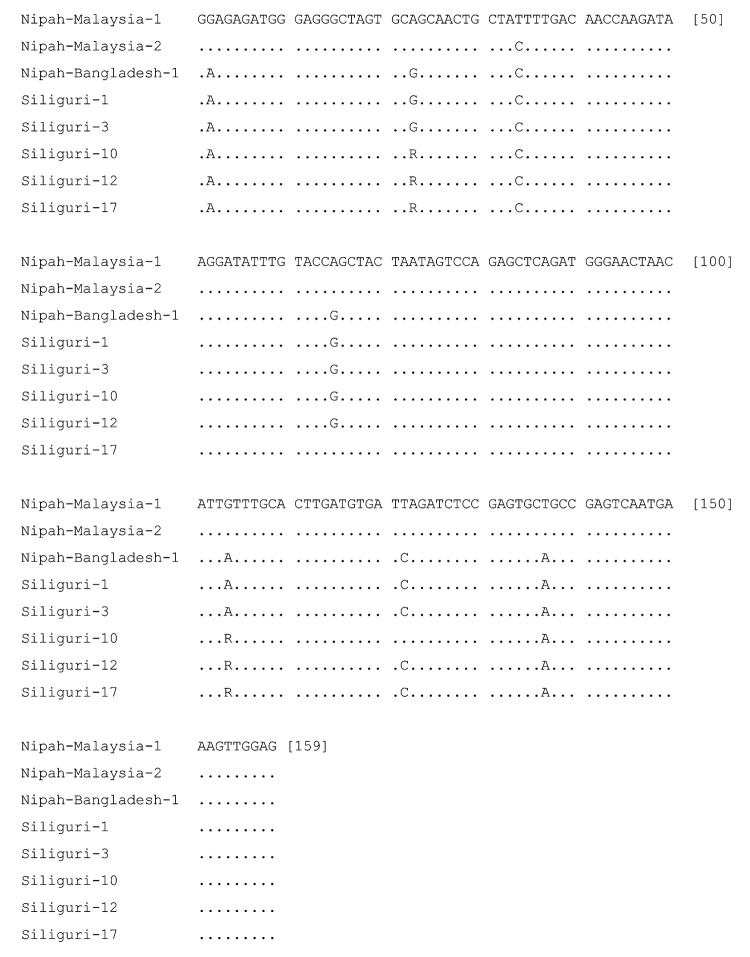
Comparison of partial N-gene nucleotide sequences obtained from the Siliguri specimens (by patient number, see Table) to sequences obtained from Nipah virus isolates from Bangladesh (AY988601) and Malaysia (AF212302, AF376747). Letters indicate positions that differ from the reference sequence on the top line, Nipah-malaysia-1. Dots indicate nucleotide identity. R indicates A or G.

**Figure 4:**
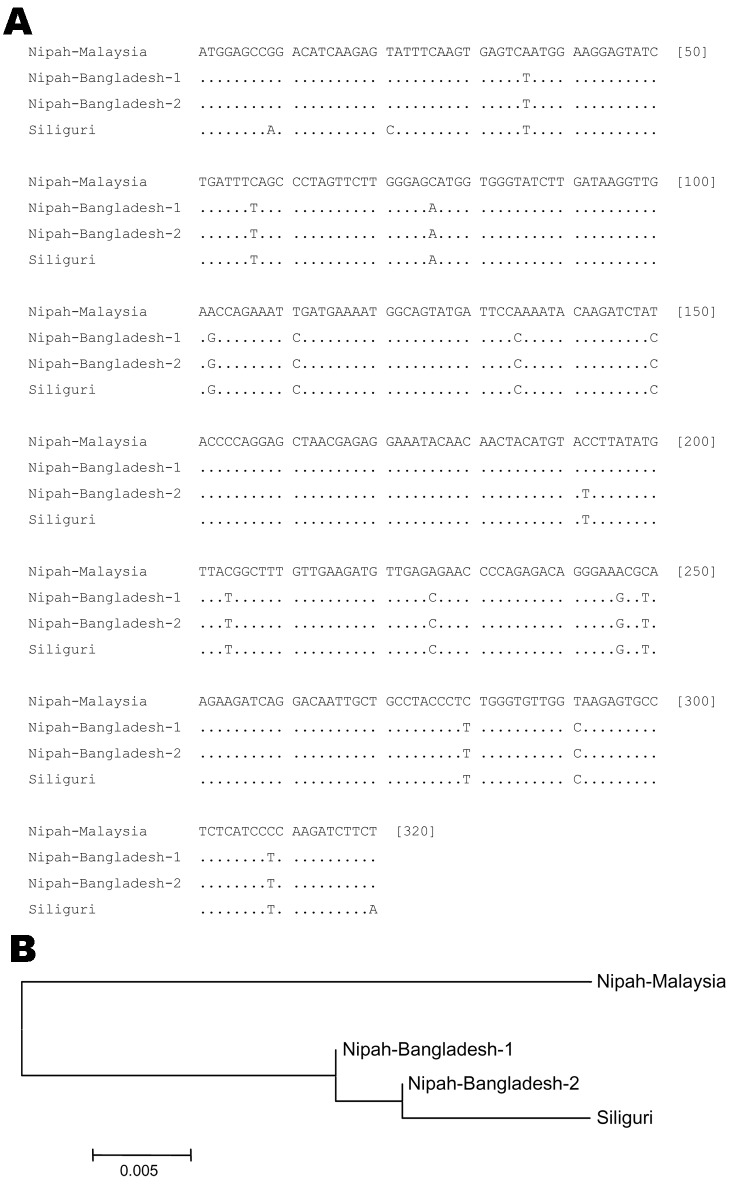
A) Comparison of partial M-gene nucleotide sequences of Siliguri specimens to Nipah virus isolates from Bangladesh (Bangladesh-1:AY988601, Bangladesh-2:unpublished) and Malaysia (AF212302). Letters indicate positions that differ from the reference sequence on the top line, Nipah-Malaysia. Dots indicate nucleotide identity. B) Phylogenetic tree based on the sequence alignment shown in panel A.

Sequence analysis confirmed that the PCR products were derived from NiV RNA (Figures 3 and 4). Partial N-gene sequences (159 nt) from 2 of 5 Siliguri samples were identical, and the other 3 sequences differed by no more than 1 nt, although unresolved sequence heterogeneity occurred at 2 positions (A or G) in 3 of the Siliguri N-gene sequences (Figure 3). Comparison of the Siliguri N-gene sequences to the N-gene sequences from NiV samples isolated in Bangladesh in 2004 and Malaysia in 1999 showed an overall level of nucleotide identity of 97.5%. Siliguri N-gene sequences were more closely related to the N-gene sequence from the Bangladesh isolate than to the sequences from the Malaysian isolates. Two of the Siliguri N-gene sequences were identical to the Bangladesh N-gene sequence.

Comparison of the partial M gene sequence amplified from the specimens from Siliguri to the M gene sequences from NiV isolated in Malaysia and Bangladesh (Figure 4) showed identity at 302 (94%) of 320 nt positions. Again, the Siliguri M gene sequences were more closely related to the M gene sequences from Bangladesh (99% identity) than to the sequences from Malaysia (94% identity).

## Discussion

This retrospective study provides evidence of NiV infection during a 2001 outbreak of febrile encephalitis in Siliguri. Nine of 18 of the patients tested had IgM and IgG antibodies; 1 had IgG antibodies only to NiV. Urine samples from 4 of these patients contained NiV RNA. One other patient had NiV RNA in the urine but lacked a detectable IgM and IgG response. In this case, the serum sample may have been obtained early in infection before antibodies to NiV were present. These laboratory results, along with the observation that the symptoms in the Siliguri patients were consistent with those described for patients during NiV outbreaks in Bangladesh and Malaysia (*3**–**5**,**15**–**17*), provide strong evidence that NiV caused the outbreak in Siliguri. Failure to detect evidence of NiV or NiV-specific antibodies in some patients may have been due to early sample collection or to inclusion of encephalitides of other causes because of the broad case definition. One patient was IgG-positive but had no detectable IgM, which suggests past infection by NiV. Unfortunately, because no case control and population-based studies were undertaken during this outbreak, interpreting this result is difficult.

The main reservoir for NiV is believed to be fruit bats of the genus *Pteropus*. NiV was isolated from fruit bats in Malaysia and Cambodia, and seropositive bats have been detected in other parts of Southeast Asia (*7**–**10*). In the Malaysian outbreak, commercially raised pigs were believed to be intermediate hosts. Presumably, the pigs were infected by virus shed from fruit bats and then transmitted the virus to humans. Although fruit bats with antibodies to NiV were captured in the outbreak areas of Bangladesh, no intermediate animal host was identified. In Bangladesh, NiV might have been transmitted to humans by direct contact with bats or indirectly by contact with material contaminated by bats. Person-to-person spread was also noted during the 2004 NiV outbreak in Faridpur, Bangladesh (*4**,**5*). The range of *Pteropus giganteus*, one of the flying foxes commonly found in south Asia (*18*), includes West Bengal. Therefore, the range of the proposed natural reservoir for NiV extends into northeastern India, and since the geographic features of West Bengal are similar to those of Bangladesh, environmental circumstances that favor transmission of NiV to humans would likely also be found in West Bengal.

Many of the epidemiologic features of the outbreak in Siliguri were similar to those of the recent NiV outbreaks in Bangladesh. In Bangladesh, no intermediate animal host was identified, whereas in Siliguri studies to detect an intermediate host were not conducted. In Siliguri, no samples were obtained from local wildlife or domestic animals. In both outbreaks, transmission occurred in healthcare settings through contact with infected persons. In Siliguri, the observation that only adults were affected supported the nosocomial transmission theory, as the number of children on the wards of hospitals was minimal. During infection, NiV is present in respiratory secretions and urine (*19*) and in both outbreaks, failure to use personal protective equipment probably contributed to the spread of the virus. Many of the patients had nasogastric tubes inserted or were intubated, procedures which made exposure to respiratory secretions more likely. Initiating adequate barrier nursing techniques helped to curtail further spread of infection.

Sequence analysis of PCR products confirmed NiV RNA. Unfortunately, no virus was isolated, and only limited sequence data could be obtained from the available clinical material. Analysis of the limited sequence data suggested that the NiV strains associated with the outbreak were more closely related to NiV isolated in Bangladesh than to NiV isolated in Malaysia. These data extend the previous observation that viruses circulating in different areas have unique genetic signatures (*10**,**14*) and suggest that these strains may have co-evolved within local natural reservoirs.

To our knowledge, NiV infection has not occurred previously in India; however, given the proximity of Siliguri to the areas of Bangladesh that experienced NiV outbreaks in 2001, 2002, and 2004, the outbreak is not surprising. Given the distribution of the locally abundant *P. giganteus*, the apparent natural reservoir of NiV in this area, outbreaks of NiV will likely continue to occur in Bangladesh and northern India. Establishing appropriate surveillance systems in these areas will be necessary so that NiV outbreaks can be detected quickly and appropriate control measures initiated. When NiV infections are suspected, infection control practices must be strengthened to avoid outbreaks in hospital settings, as apparently occurred in Siliguri.
